# Predicting lithium metal anode failure at an early stage

**DOI:** 10.1093/nsr/nwaf248

**Published:** 2025-06-17

**Authors:** Jian Liang Cheong, Zhi Wei Seh

**Affiliations:** Institute of Materials Research and Engineering (IMRE), Agency for Science, Technology and Research (A*STAR), Singapore; Institute of Materials Research and Engineering (IMRE), Agency for Science, Technology and Research (A*STAR), Singapore

**Figure 1.**
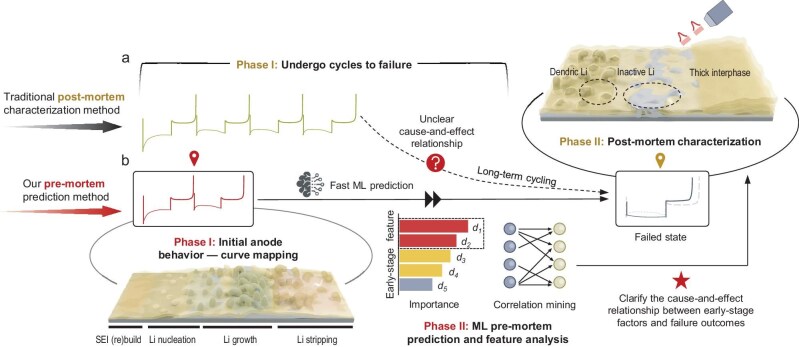
Schematic of (a) the traditional post-mortem characterization method for studying failure mechanisms and (b) the pre-mortem prediction method used in this study. The initial-two-cycle features serve as early-stage electrochemical fingerprints for predicting failure types. Reproduced with permission from [3].

Electrochemical curve fingerprints, coupled with machine learning (ML), offer a powerful new approach to understanding and predicting the failure mechanisms in lithium metal anodes—a critical component for advancing lithium metal batteries [1,2]. Unlike traditional post-mortem analysis, which only reveals the end state of failure, Guangmin Zhou *et al.* reported a novel pre-mortem prediction method that led to the identification of the degradation root cause of early-stage electrochemical behaviours (Fig. 1) [3]. Leveraging on a vast dataset of >18 000 cycles and 12 million data points from failed battery cells, a correlation between the initial lithium plating/stripping behaviour and subsequent anode changes was established. The correlation was manifested as an ML model, which accurately predicts failure types by using data from only the first two cycles (<2% of the total data), enabling the early identification of failure indicators and accelerating electrolyte optimization. The study categorizes failures into kinetics degradation failure (KDF), reversibility degradation failure (RDF), and co-degradation failure (CDF) based on changes in the overpotential (*α*) and coulombic efficiency, with each type exhibiting a unique curve evolution pattern. Building on this classification, analysis of the feature importance ranking from the ML model revealed that highly ranked features encompass the lithium nucleation and thickening processes. Analysis of the feature importance of the ML model revealed that lithium nucleation and thickening processes are critical to the failure type. Highly ranked nucleation kinetics features (d1, d4, d6) are linked to solid–electrolyte interphase (SEI) properties [3]. For example, KDF-type failures showed significantly lower relative nucleation overpotential (d1), consistently with better SEI ionic conductivity and lower Young's modulus. The study also detailed how SEI properties (composition, mechanical strength) influence initial plating/stripping behaviours [3]. For instance, a CDF-type SEI, rich in organic components, showed poor ion transport and mechanical strength, while the RDF type, with high LiF content, exhibited high mechanical strength but suboptimal ion conductivity. Additionally, finite element analysis and scanning electron microscopy allow observation of the lithium-thickening-process transitions from porous vertical to dense lateral growth, leading to an increase in the local current density and SEI impedance. This raises overpotential and can be attributed to changes in the effective lithium-ion transfer area within the SEI. Taken together, these insights directly connect early-stage electrochemical fingerprints to underlying SEI properties and their influence on battery-failure mechanisms. Post-failure structural analysis, carried out by using electron microscopic techniques [3], determined that CDF involved inactive lithium and irreversible SEI impedance from dendritic growth. The high SEI modulus of the RDF caused fracture, porous ineffective SEI and surface inactive lithium. KDF, having a moderate SEI modulus, formed dense ineffective SEI, hence increasing the migration impedance. These results confirm that early-stage factors dictate these pathways, impacting charge transport.

To conclude, the pre-mortem prediction method developed by Guangmin Zhou *et al*. offers significant advantages over traditional post-mortem analysis, enabling faster battery reliability assessment and accelerating electrolyte development by uncovering degradation root causes from initial cycles. This allows informed decisions on battery suitability, streamlining the development of advanced electrolyte formulations for high-performance lithium metal batteries. The methodology also holds potential for extension to other metallic anodes such as sodium [4] and zinc [5].
